# The role of *Rhodotorula mucilaginosa* in selected biological process of wild fish

**DOI:** 10.1007/s10695-018-0591-0

**Published:** 2018-12-05

**Authors:** Elżbieta Bogusławska-Wąs, Alicja Dłubała, Maria Laskowska

**Affiliations:** Department of Applied Microbiology and Biotechnology, Faculty of Food Science and Fisheries, West Pomeranian University of Technology, Papieza Pawla VI 3, 71-450 Szczecin, Poland

**Keywords:** Yeasts, *Rhodotorula*, Fish, EPS, Carotenoid pigments

## Abstract

Defense mechanisms of fish are investigated in many aspects. One of the most interesting systems is that based on non-specific immune factors whose mechanisms of biocontrol have evolved in complex processes of microbiological co-existence. The wild fish devoid of probiotic stimulation have developed their own system to control the biosynthesis of immunostimulating compounds based on commensal microflora. Results of this study demonstrated the gastrointestinal tract (GI) of wild fish (*Abramis brama*, *Rutilus rutilus*, *Perca fluviatilis*) was colonized by permanently residing strains of *Rhodotorula mucilaginosa*. The genetic profile of the tested strains (PCR–random amplification of polymorphic DNA) indicated their affinity only to the GI of the analyzed fish. The capability for biosynthesis of β-carotene, torulene, torularhodin, and exopolysaccharides (EPS) under conditions of fish gastrointestinal tract was found to be a strain-specific trait. *Rhodotorula* spp. interactions with fish should be considered as a mechanism of symbiotic relations based on the stimulation of non-specific mechanisms of fish immunoprotection and antioxidative properties of yeast.

## Introduction

The knowledge on the ecological role of microflora of wild fish gastrointestinal tract, especially that associated with yeast prevalence, is still insufficient. An important study area includes identification of the natural composition of this microflora, as the co-relationships within it may allow determining the real role of yeast in vital processes of fish. The functionality of unicellular fungi does not change in time on condition that they represent an element of permanent microbiota. In most cases, their counts do not exceed 2.0–5.0% of the total population of microorganisms isolated from fish. In the case of healthy and cultured fish, yeast prevalence is mainly discussed as closely related to the mechanisms of immunostimulation and to the process of gastrointestinal tract maturation (He et al. 2011; Gopalakannan and Arul 2010; Meena et al. 2013). As yeast represent a valuable source of enzymes, vitamins, amino acids, and oligosaccharides (Lokesh et al. 2012), in the opinion of some scientists, they are better growth promoters than the probiotic bacteria are (Caruffo et al. 2015). Their antimicrobiological properties are also increasingly often emphasized (Hatoum et al. 2012). Investigations on the health-promoting effects of yeast concern mainly *Saccharomyces cerevisiae*, *Debaryomyces hansenii*, and *Phaffia rhodozyma* (Caruffo et al. 2015; Irianto and Austin 2002) have been mainly conducted with cultured fish, while the aspect of the ecological role of yeast in wild fish seems to be completely neglected.

The prevalence of yeast in the biosphere is not random. It is subject to biological regulation by communities producing them and depends on preferences for the colonized site (Boguslawska-Was 2010). A thesis was adopted in this study that yeasts—as opportunistic pathogens—do not pose any direct health risk and influence the regulation of fish defense mechanisms. It was assumed that metabolites of physiologically active yeast may be used by fish in multiple biological processes. The functionality of unicellular fungi does not change if that they represent an element of permanent microbiota and that homeostasis is maintained between microorganisms, which ensures keeping the safety rigor. Hence, in this work, we have focused only on these yeast strains whose prevalence was not prevented by conditions of the gastrointestinal tract environment.

## Materials and methods

### Sample collection and isolation of yeast

Samples of water and three fish species, bream (*Abramis brama* Linnaeus, 1758), roach (*Rutilus rutilus* Linnaeus, 1758), and perch (*Perca fluviatilis* Linnaeus, 1758), were collected from March to November from three zones of Odra River Estuary. The above fish species were selected for the study based on traits of their gastrointestinal tract, feeding area, and place in the trophic chain. Their gastrointestinal tract (GI) and epidermal mucus (EM) were examined. In total, 120 fish and 30 water samples were analyzed in the study. Microbiological analyses were carried out on the sampling day. The GI of fish was prepared aseptically, intestinal digesta was removed, and then the tract was flushed and homogenized in physiological saline solution (0.9% *v*/*w*, pH 7.0). Mycological analyses of GI and EM were conducted on the SDA culture medium (Sabouraud dextrose agar 0.5%—yeast extract, 2.0%—glucose, 2.0%—agar; pH 4.5; Scharlau) with the addition of chloramphenicol (Interforum Pharma). Incubation was conducted at a temperature of 22 °C for 48–72 h. In turn, microbiological analyses of water samples were conducted using a membrane inoculation technique (Sartorius) under culture conditions as described above.

### Biochemical and genetic characterization of the isolates

Strains were identified based on their macroscopic and biochemical traits using the API 20C AUX system (bioMerieux). Species identity of selected strains (*Rhodotorula mucilaginosa* and *Rhodotorula glutinis*) was confirmed with the PCR method, accordingly to White et al. (1990). Cultures were incubated on the YEPD medium (1.0% yeast extract, 2.0%—peptone, 2.0%—glucose; Scharlau) at 22 °C for 24 h. Genetic differentiation analysis was carried out with the random amplified polymorphic DNA method (RAPD-PCR). Genomic DNA was isolated following the protocol of the QIAamp DNA Mini Kit (Qiagen) using lyticase (Sigma-Aldrich). Strains of *Rh. mucilaginosa* were differentiated with the following combination of starter cultures: 1254: CCg CAg CCA A and 1290: gTg gAT gCg A, whereas *Rh. glutinis* intraspecies differentiation was conducted with ERIK2: AAgTAAgTgACTggggTgAgCg (Bogusławska-Wąs 2010). The RAPD-PCR reaction was conducted in 25 μL of the reaction mixture containing the following: 500 mM KCl, 100 mM Tris–HCl (pH 8.3 at 25 °C), 1.25 mM MgCl_2_, 0.3 mM dNTP, 20 pmol/μL of each starter, 1 U Taq DNA polymerase (Eppendorf), and 20 ng DNA template. The thermal profile used in the reaction consisted of 42 cycles, including 60 s/94 °C, 60 s/36 °C, and 60 s/72 °C. Amplification products were separated electrophoretically in 2.0% agarose gel (Prona Agarose Plus) with ethidium bromide (0.5 μL/mL) (Bio-Rad, USA). Results of the electrophoretic separation were visualized in UV rays in a Gel Doc apparatus (Bio-Rad). Strain affinity degree was determined based on cluster analysis of the obtained amplification profiles (RAPD) with the unweighted pair group method with arithmetic mean (UPGMA) method in Bio-Gene software (Vilber Lourmat), with Dice coefficient of 3.0%.

### Microbial adherence

Adherence of yeast strains was determined with the method of tetrazolium salt reduction (XTT; Sigma-Aldrich) accordingly to Jin et al. (2004). The experiment was conducted on 96-well titration plates (Sarstedt). Before being used, the plates were coated with 3% BSA (100 μL/well) and left overnight at a temp. of 4 °C. Next, the excess of bovine serum albumin was decanted, and the plates were double-rinsed with HEPES (pH 7.0). The suspension of yeast strains (100 μL) was introduced to wells and incubated at a temp. of 22 °C. To determine cell adherence degree (after 2, 6, 12, 24, and 48 h of incubation), the excess of culture was removed and the wells were double-rinsed with PBS. Afterwards, 100 μL of the reaction mixture containing 1580 μL PBS, 200 μL XTT, and 20 μL menadione (2-methyl-1,4-naphthoquinone; Sigma-Aldrich) was added accordingly to modified method of Ramage et al. (2001). The plates were incubated at 22 °C/2 h, and then absorbance of the supernatant was measured at *λ* = 490 nm (Antachopoulos et al. 2006). Measurements were made with the Elx808 reader (BioTek Instruments) (Balcazar et al. 2008).

### Cell growth and EPS production kinetics

Mineral medium (SM) with pH 5.6 and the following composition was used to culture the test strains: 0.1% yeast extract, 0.2% (NH_4_)_2_SO_4_, 0.1% KH_2_PO_4_, 0.05% MgSO_4_, 0.01% CaCl_2_, 0.01% NaCl, and 5% sucrose (Ghada et al. 2012). Inoculum (5.0% of total medium volume) consisted of 48 h yeast cultures prepared as above. The cultures were shaken (200 rpm) at a temp. of 22 °C for 72 h. Changes in cell counts were monitored by OD measurements at *λ* = 550 nm (NanoDrop ND-1000). The concentration of exopolysaccharides was determined with the ethanol precipitation method (Pavlova et al. 2005). A 50-mL sample was collected from the cultures and centrifuged in extraction flasks (6000×*g*/30 min). Supernatant was collected, and 96% ethanol was added (at 1:2 ratio). To obtain the precipitate, the samples were stored at 4 °C for 24 h, and then centrifuged (6000×*g*/10 min). The resultant precipitate was rinsed with ethanol, centrifuged again, and dried until dry weight has been reached. Results obtained were used to calculate the specific production coefficient (*Y*_*p*/*x*_), accordingly to the following formula:

$$ {Y}_{p/x}=P/x $$

where *P*—maximum exopolysaccharide concentration and *x*—yield of dry biomass.

### Carotenoid pigments

Biosynthesis of carotenoid compounds was determined in 72 h cultures which were incubated in the SM medium at a temp. of 22 °C and shaken at 200 rpm. Culture sample (10 mL) was then centrifuged (6000×*g*/10 min). The resultant precipitate was double-rinsed with distilled water and centrifuged again. Afterwards, DMSO (5 mL at 55 °C) was added and the sample was vortexed (60 s). Next, hexadecane and 20% NaCl were added in series (0.5 mL) (Stachowiak 2012). Absorbance of total carotenoids was measured with a UV–VIS spectrophotometer at *ƛ* = 490 nm (Cheng and Yang 2016). In the case of torulene and β-carotene, measurements were carried out at *λ* = 485 nm and *λ* = 450 nm, respectively. The total content of carotenoids was calculated acc. to Maldonade et al. (2012) assuming the absorbance coefficient of *A*^%^_1 cm_ = 3240 and expressed in micrograms per gram of dry matter.

## Results

### Differentiation of yeasts

Mean counts of yeast isolated from water samples did not indicate any significant (*p* > 0.05) differences between seasons of the year and did not exceed 2.4 log cfu 100 mL^−1^. In the case of fish mycocenoses, the level of GI colonization by unicellular fungi was comparable. The numbers of fungi determined for all fish species ranged from 2.44 to 2.94 log cfu g^−1^. The significance of differences was not confirmed in any case (*p* < 0.05). In contrast, the number of yeast isolated from EM of all fish in the summer season was statistically lower (2.24–2.63 log) than in the spring and autumn seasons (3.55–4.43 log and 3.68–4.51 log, respectively). Results of these analyses are presented in Table 1.

**Table 1:** Total count of yeasts isolated from fish and sampling water

Samples	Origin	Number of fish or water tested	Spring	Summer	Autumn
			Log cfu g^−1^·100 mL^−1^*		
Water	EO	30	1.93 (± 2.01)	2.33 (± 1.75)	2.36 (± 2.33)
*Abramis brama*	EM	40	3.55 (± 3.01)	2.24 (± 1.72)	3.68 (± 2.65)
	GI		2.78 (± 2.29)	2.83 (± 2.21)	2.94 (± 2.43)
*Rutilus rutilus*	EM	40	4.43 (± 4.27)	2.27 (± 1.79)	4.45 (± 3.25)
	GI		2.44 (± 2.28)	2.61 (± 2.47)	2.66 (± 1.77)
*Perca fluviatilis*	EM	40	4.23 (± 3.95)	2.63 (± 2.24)	4.51 (± 3.96)
	GI		2.49 (± 2.01)	2.92 (± 1.75)	2.57 (± 2.33)

Results are means ± SD

*EM* epidermal mucus, *GI* gastric intestinal

*Samples of water

### Collection of samples and mycological analyses

Of the 22 species of unicellular fungi isolated from fish catching sites, 10 were also detected in the ichthyofauna. Repeatedly isolated from all analyzed habitats were representatives of the following genera from the Basidiomycetes classes: *Cryptococcus* (*Cr. uniguttulatus*) and *Rhodotorula* (*Rh. glutinis* and *Rh. mucilaginosa*). The quantitative and qualitative analyses showed *Rh. mucilaginosa* to predominate in the mycocenotic structure. Its contribution in the total number of yeast isolated from EM was determined at 19.5% in bream, 23.1% in roach, and 19.05% in perch. In turn, the contribution of *Rhodotorula* spp. in GI samples was at 20.0%, 17.9%, and 41.7%, respectively (Table 2).

**Table 2:** Occurrences of yeasts (%) in samples from fish

Species	Water		*Abramis brama*				*Rutilus rutilus*				*Perca fluviatilis*			
			EM		GI		EM		GI		EM		GI	
*C. albicans*	1.5	op	0.0	–	0.0	–	0.0	–	0.0	–	0.0	–	0.0	–
*C. colliculosa*	0.2	op	4.0	ci	5.7	op	2.0	ci	8.0	op	1.3	op	0.0	–
*C. famata*	5.6	ci	5.3	ci	9.7	op	11.6	ci	12.0	op	10.6	ci	4.3	op
*C. guilliermondii*	0.6	op	1.1	op	6.3	op	3.9	op	3.7	op	3.6	ci	0.7	op
*C. holmii*	1.1	op	0.3	op	5.0	op	1.3	op	2.3	op	0.6	op	0.0	–
*C. lipolytica*	2.2	ci	0.0	–	0.0	–	1.0	op	6.0	op	0.0	–	0.0	–
*C. melibiose*	0.5	op	0.7	op	0.0	–	2.6	op	0.0	–	1.3	op	0.0	–
*C. membranifaciens*	1.8	op	0.0	–	0.0	–	1.2	ci	4.3	op	0.6	ci	0.7	op
*C. sake*	2.5	op	2.7	op	0.0	–	4.0	op	2.3	op	2.0	op	0.0	–
*C. tropicalis*	0.7	op	0.0	–	0.0	–	0.0	–	0.0	–	0.0	–	0.0	–
*Cr. albidus*	5.6	ci	5.0	ci	0.0	–	0.0	–	5.0	op	0.0	–	0.0	–
*Cr. humicolus*	1.0	op	0.7	op	0.0	–	0.0	–	0.0	–	0.0	–	0.0	–
*Cr. laurenti*	6.0	ci	5.3	ci	6.0	ci	2.1	op	7.0	op	4.5	op	5.0	op
*Cr. neoformans*	5.6	op	0.0	–	0.0	–	0.0	–	0.0	–	0.0	–	0.0	–
*Cr. uniguttulatus*	10.3	ci	17.5	ci	12.3	ci	17.6	ci	7.0	ci	14.9	ci	7.0	ci
*Kl. apiculata*	1.1	op	0.0	–	0.0	–	0.0	–	0.0	–	0.0	–	0.0	–
*P. carsonii*	3.7	ci	10.3	ci	8.3	op	5.0	ci	6.7	op	16.7	ci	11.3	ci
*Rh. glutinis*	18.3	ci	12.0	ci	9.0	ci	20.5	ci	5.0	ci	20.1	ci	15.8	ci
*Rh. minuta*	5.0	op	6.0	op	5.3	op	2.9	op	6.0	op	1.6	op	8.2	op
*Rh. mucilaginosa*	14.9	ci	19.5	ci	20.0	ci	23.1	ci	17.9	ci	19.5	ci	41.7	ci
*S. cerevisiae*	11.1	ci	4.6	ci	7.7	op	1.6	op	6.8	op	1.6	op	0.3	op
*Tr. mucoides*	0.4	op	4.0	ci	4.7	op	0.0	–	0.0	–	1.1	op	0.0	–

*ci* constantly insulated, *op* occasionally insulated

### Genetic diversity of *Rh. mucilaginosa*

Yeast strains permanently isolated and predominating in the species structure of all analyzed habitats were subjected to interspecies typing with the RAPD-PCR method. Three non-affiliated genetic lines were distinguished based on the genetic patterns of *Rh. mucilaginosa* (*S* < 50% at Dice 3%), within which genotypes of strains with close affinity were distinguished at *S* > 75% (Table 3). Genotypes I—AB, I—BA (except for EM *R. rutilus*), and I—BC (not detected only in fish GI) were isolated from all analyzed environments. In the case of genetic lines II—A and B and genotypes I—AA and BB, the isolates originated exclusively from the aquatic environment. The most diverse profile of the obtained RAPD patterns was noted for the group III—A and B (Fig. 3). The grouped strains were isolated exclusively from GI. Strains with genotypes III—AB and AC were found only in bream, whereas these with genotype III—AA were isolated only from perch. There was no *Rh. mucilaginosa* genotype that would be typical only of roach (Table 3).

**Table 3:** The occurrence of *Rh. mucilaginosa* genotypes

Genetic line		Genotype						
*S* < 50%	50.0% < *S* < 75.0%	*S* > 75.0%						
		Water	*Abramis brama*		*Rutilus rutilus*		*Perca fluviatilis*	
		EO	EM	GI	EM	GI	EM	GI
I	A	AA	–	–	–	–	–	–
		AB	AB	AB	AB	AB	AB	AB
	B	BA	BA	BA	–	BA	BA	BA
		BB	–	–	–	–	–	–
		BC	BC	–	BC	–	BC	–
II	A	AA	–	–	–	–	–	–
		AB	–	–	–	–	–	–
	B	BA	–	–	–	–	–	–
		BB	–	–	–	–	–	–
III	A	–	–	–	–		–	AA
		–	–	AB	–	AB	–	–
		–	–	AC		–	–	–
	B	–		BA	–	–	–	–

### Microbial adherence

**Fig. 1:**
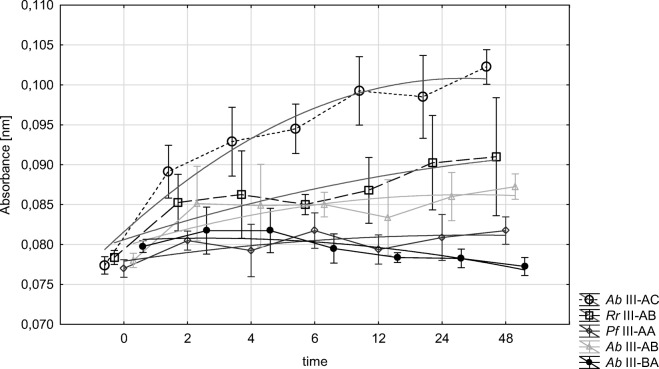
Adhesion of the yeast genotype

For further analyses, we used only isolates typical of GI, i.e., strains of *Rh. mucilaginosa* with genotypes III: AB, AC, BC, and AA. Adherence kinetics and biofilm formation were determined via colorimetric determination of XTT reduction, as an indicator of the metabolic activity of cells. The strains isolated from fish GI were found capable of biofilm formation, except for the genotype III—BA. In the other cases, cell density increased with time and reached *plateau* after 6–12 h (Fig. 1). The progress in cells adherence was correlated with the genetic profile of strains, which was confirmed in the statistical analysis (*p* < 0.05). There was no significant correlation between strain behavior and fish species.

### EPS production kinetics

**Fig. 2:**
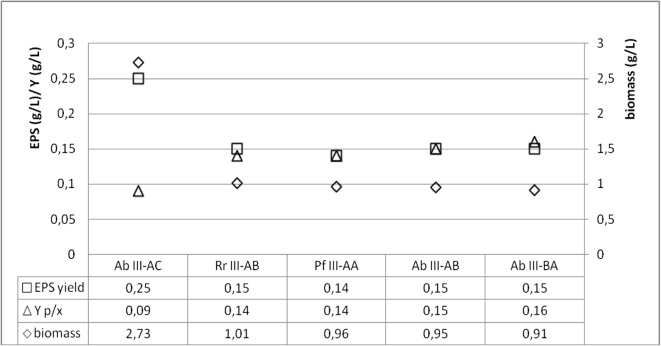
EPS production as a function of cellular growth

Results of the statistical analysis demonstrated significant differences in biomass yield and EPS concentration produced in cultures of *Ab* III—AC genotype strains (2.73 g/L and 0.25 g/L, respectively). In the remaining cultures, the respective values were comparable and fitted within the following ranges: 1.01–0.91 g/L and 0.14–0.15 g/L. In addition, no significant differences were determined in the production of the analyzed polymers between strains of the distinguished genotypes. Their concentration was proportional to biomass production. For this reason, we calculated the specific production coefficient (*Y*_*p*/*x*_) which is indicative of the real EPS productivity by 1 g of biomass. The mean *Y*_*p*/*x*_ value ranged from 0.14 to 0.15 in the case of the strains with genotypes III—AB, AA, and AC and was almost twofold higher than the coefficient calculated for the strains with III—AC genotype, i.e., 0.09 (Fig. 2).

### Biosynthesis of carotenoids by *Rh. mucilaginosa*

Spectrophotometric multi-component analysis was used to quantitate carotenoids synthesized by *Rh. mucilaginosa* strains. Results of absorbance measurements demonstrated a correlation between strain genotype and their capability for pigments biosynthesis. Significant deviations noted in the values obtained for individual strains with the distinguished genotypes reflected also the strain-specific character of this capability. The highest carotenoid-producing capability was determined for strains with the *A. brama* III—AB genotype, whereas the lowest ones for these with the *A. brama* III—BA genotype (Table 4). Results of absorbance measurements point to the presence of a combination of three pigments—β-carotene, torularhodin, and torulene (Table 4). All analyzed strains produced a similar quantity of β-carotene. The content of torularhodin fraction in the total carotenoid content ranged from 25% for strains with *A. brama* III—BA genotype to 48.9% for these with *P. fluviatilis* III—AA genotype, whereas that of torulene reached 22.7% for *R. rutilus* III–AB strains and 46.5% for *A. brama* III—BA strains (Table 4).

**Table 4:** Carotenoids produced of *Rh. mucilaginosa*

Carotenoid production	Genotype				
	*A. brama* III—AC	*R. rutilus* III—AB	*P. fluviatilis* III—AA	*A. brama* III—AB	*A. brama* III—BA
Total carotenoid content (μg g^−1^)	159.4 (± 46.963)	183.8 (± 53.418)	149.1 (± 66.184)	226.83 (± 53.195)	131.2 (± 41.033)
Pigment composition (%)					
β-Carotene	27.9	30.3	27.1	27.0	28.5
Torulene	38.4	47.0	48.9	36.8	25.0
Torularhodin	33.7	22.7	24.0	36.2	46.5

Results are means ± SD

## Discussion

Fish colonization by yeast may have a significant impact on changes in relations between the host and the developing microbiota (Ringo et al. 2003). The first and key element of colonization control is the epidermal mucus. Its immunoglobulins, lectins, lysozyme, antimicrobial polypeptides, and compounds of various polarities regulate processes of biocontrol targeted mainly at pathogenic bacteria. The development of pathogenic microflora is often accompanied by the effect of superinfection with yeast-like fungi (Tartor et al. 2018). Even a minimal amount of lysozyme can inhibit the reproduction of some yeast. Such mechanisms are believed to stem from ultrastructural and morphological changes of budding forms (Lopera et al. 2008). The effect of seasonal variability of yeast number in mucus is, thus, correlated with the physiological condition of a fish and its response to changes in environmental conditions. In the winter–spring period (December–April), a significant decrease is observed in the content of, e.g., hydrolytic protein, which is the direct reason behind increased counts of determined fungi (Table 1), and an increase in ATPases content in the epidermal layers of fish mucus (Schrock et al. 2001). It is the only justification of mycocenotic relations observed in our study between water and epidermal mucus. Comparable numbers of yeast in water samples are collected in all studied seasons (log 1.93–2.36), and their seasonal variations in EM (spring log 3.55–4.43 and autumn log 3.68–4.51) were not correlated with GI mycocenoses of the investigated fish (log 2.44–2.94). It was concluded that results of the qualitative and quantitative differentiation of yeast cannot be due only to their passive ingestion with feed. The analyzed fish species (bream, roach, perch) were characterized by a common feeding area—soft bottom and meadows of seagrass—and by diverse feed preferences (Elliott and Hemingway 2002). For this reason, we speculated that some of the yeasts could be natural symbionts.

When investigating the biological function of yeast in fish physiology, an overriding stage is to identify species claimed to be permanent residents. Of the 23 species isolated from water and fish samples, only three, *Cr. uniguttulatus*, *Rh. glutinis*, and *Rh. mucilaginosa*, appeared to be isolated permanently, with *Rh. mucilaginosa* having the greatest contribution in the mycocenotic structure. Of the isolated *Rhodotorula* strains, we selected these with genetic profiles typical only of the gastrointestinal tract of fish. Selected at the level of *S* < 50%, as strains non-affiliated with representatives of *Rh. mucilaginosa* isolated from water and mucus, they constituted a separate genetic group. Their intragroup affinity, determined at *S* > 75%, allowed us to conclude that they represent an integral part of the microbiota of fish GI. Considering the above, we hypothesized about the capability of *Rhodotorula* spp. for permanent colonization of the gastrointestinal tract. Qualitative and quantitative differences in the microbiocenosis are determined by microbiological colonization at the early stage of fish development as well as by environmental conditions and changes in fish diet (Ringo et al. 2006; Olafsen 2001; Caruffo et al. 2015). The survival strategy of microorganisms is based on their adherence to GI mucus and using its components as sources of nutrients and energy. This explains typing strains of *Rh. mucilaginosa* at the probability level of *S* > 75% and their permanent residence in the GI. Among the four distinguished genotypes of *Rh. mucilaginosa*, only the strains grouped in the BA genotype did not show the capability to adhere to GI mucus (Fig. 1), despite comparable rate of biomass formation and production of EPS as a biofilm stabilizer (Fig. 2). The kinetics of biofilm formation was the highest in the case of the AC genotype strains (Fig. 1). Worthy of notice, however, is the fact that this resulted from the rate of cell reproduction and not from the traits being indicative of the outstanding productivity of extracellular compounds (Fig. 2).

In the fish-yeast symbiotic regulation, the specific adherence of yeast is also aided by the presence of glycoprotein mucins secreted mainly by the epithelial cells of the gastrointestinal tract. They are composed of a high number of serine residues whose presence affects the process of fungi adherence. The colonization of GI by *Rhodotorula* spp., supported by the natural mechanisms of fish, alters the environment surrounding them. Metabolites produced by yeast modify physicochemical properties of the environment, and together with it, they induce processes of microbiological control. Synergistic dependencies between the surface of their cell wall and the epithelium are stabilized by extracellular compounds (EPS) synthesized by *Rhodotorula*. Overproduction of these polymers, as a consequence of activated defense mechanisms, reflects responses induced by the cellular stress. These responses are not limited only to interactions with EPS producers (yeast cells) but are becoming a protective barrier for GI ichthyofauna by being a matrix for an epibiotic biofilm development (Boguslawska-Was 2010). One of the biological functions of EPS is their capability to inhibit the growth of bacteria and to, simultaneously, suppress lysozyme activity towards yeast. This presumption was confirmed by results of our study (Fig. 2). The fact of extracellular biopolymers formation should be interpreted in connection with the possibility of controlled cellular reproduction of *Rhodotorula*. Parameters of specific productivity (*Y*_*p*/*x*_) computed for all analyzed genotypes show explicitly that EPS biosynthesis is a strain-specific trait. A lack of a strict correlation between the biomass formation and biosynthesis of extracellular compounds may suggest the existence of control mechanisms which reduce yeast prevalence in GI. EPS which are polysaccharides constitute a very good source of nutrients (Chapot-Chartier 2014; Hou et al. 2013; Molina et al. 2012). Their composition is claimed to be responsible for prebiotic properties (Navarrete and Tovar-Ramírez 2014) and for the stimulation of glucan activity in processes of immunostimulation (Tovar-Ramírez et al. 2010).

**Fig. 3:**
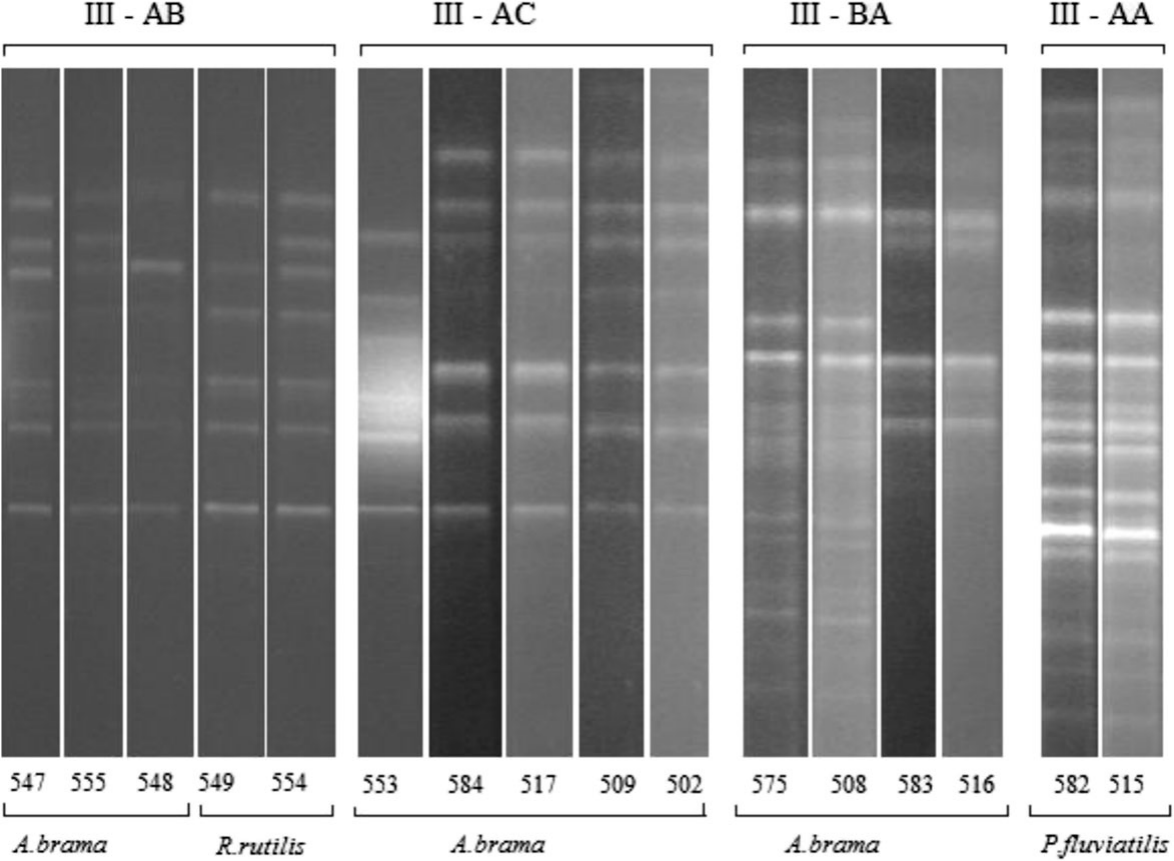
Examples for the RAPD-PCR of *Rh. mucilaginosa* isolated from GI of fish

The symbiotic role of *Rh. mucilaginosa* includes also its natural capability to synthesize carotenoids (Fig. 3). Results obtained in our study indicated no correlation between the genotype and amount of accumulated pigments. This trait was found to be strain-dependent (Table 2). In yeast cells, the carotenoids play primarily the function of membrane-protective antioxidants. However, their participation in the mechanisms of response to exogenous stress factors points to their significantly wider biological activity. Physiological demand for molecules of this type is not permanent but reflects cell adaptation to environmental changes (Cheng and Yang 2016; Marova et al. 2011). This concerns also fish which are incapable of endogenous synthesis of these pigments. It is of special significance for immune system activation processes, including activation of mechanisms controlled by non-specific protection systems. The permanent presence of *Rh. mucilaginosa* in the gastrointestinal tract may provide a source of valuable compounds used to counteract effects of oxidative stress. Its most often causes include stress and progressing infections (viral, bacterial, or parasitic). In response to threat, the immune system is stimulated and reactive oxygen and nitrogen species (ROS and RNS) are activated as molecules aiding defense mechanisms. Extreme situations may evoke overproduction of these pro-oxidants, which leads to permanent changes in host cells (Splettstoesser and Schuff-Werner 2002). According to Costantini, the effect of autodestructive changes is also ascribed to the excessive expression of stress hormones—corticosteroids, induced by outcomes of intense oxidative stress (Costantini et al. 2008). Likewise in the silencing of the excessive response of the immune system, also in the case of hormonal regulation, significant role will be ascribed to the mechanisms of release of compounds compensating for deficiencies of endogenous antioxidants. The active cells of *Rhodotorula* co-forming the commensal microbiota or accumulated products of their biosynthesis enable activation of the carotenoid system. The initiation of mechanisms enabling the release of non-enzymatic molecules that capture free radicals and cytotoxic molecules (Chew and Park 2004) contributes to the alleviation of the outcomes of the oxidative stress. Biosynthesis and circulation control of carotenoids or their derivatives used by fish (Mougeot et al. 2010) are due to the response to the host’s needs (Marova et al. 2010; Sefc et al. 2014). Results of our experiment clearly indicate that all analyzed strains, found to be the natural epibiota of the gastrointestinal tract, were producers of β-carotene, torulene, and torularhodin fractions typical of *Rh. mucilaginosa* (Table 2). It is difficult to establish which of them is more significant to fish, because the antioxidative effect of carotenoids is believed to result from the so-called overlapping activities of individual fractions. However, considering the range of the biological functions, the key role is ascribed to β-carotene (Mannazzu et al. 2015). Its production was determined at a similar level for all strains isolated from GI of the analyzed fish. However, torularhodin is much more efficient than β-carotene in protecting cells against toxic action of singlet oxygen (Sakaki et al. 2001). Determination of torularhodin and torulene contents demonstrated their varying contributions in total carotenoids. Known as vitamin A precursors and singlet oxygen radical scavengers, they are regulated by environmental factors (El-Banna et al. 2012).

Carotenoids are the main elements of the arsenal of antioxidants in animals. However, the role of *Rhodotorula* in the gastrointestinal tract should not be limited only to their natural source. Many investigations have also confirmed carotenoids involvement in the immunostimulating processes through enhanced activation of T lymphocytes, through supporting the action of macrophages (Pérez-Rodríguez 2009), or through the antimicrobial activity. Already at the end of 1990s, Calvente has formulated a thesis that siderophores secreted by *Rh. glutinis* play a significant role in processes of iron-dependent mycotic virulence (Calvente et al. 2001). Inhibition of microorganism development through the formation of ferric complexes with rhodotorulic acid of yeast results from the competition of siderophores with iron whose availability is a key element in host–bacterial flora interactions.

Yeast predispositions for colonizing, most of all, the gastrointestinal tract of fish seem obvious. The optimal pH for their growth (Martinem et al. 2006), tolerance to selective factors, and natural traits of selected *Rhodotorula* strains contribute to their classification as an element of naturally developing microbiota of fish. The biological activity of these fungi is exploited not only as a result of direct relations with the higher organism they colonize. Consortium relations between microorganisms become an element of the protective system against pathogenic microorganisms (bacteria and viruses). As a result of all these processes, the wild fish can compensate for deficits caused by fluctuations in their defense mechanisms and their response to environmental stress.
